# Investigation of Carbonic Anhydrase Inhibition, Antioxidant Properties, and Selective Anticancer Activity of Methyl‐Substituted Halogenated and Methoxy Conduritols

**DOI:** 10.1155/bmri/5819417

**Published:** 2026-02-12

**Authors:** Hayrani Eren Bostancı, Ebrar Büşra Yıldırım, Feyza Nur Çetin, Dilek Kaplan, Ümit Muhammet Koçyiğit, Alireza Poustforoosh, Burak Tüzün, Latif Kelebekli

**Affiliations:** ^1^ Department of Biochemistry, Faculty of Pharmacy, Sivas Cumhuriyet University, Sivas, Turkey, cumhuriyet.edu.tr; ^2^ Department of Chemistry, Faculty of Sciences and Arts, Ordu University, Ordu, Turkey, odu.edu.tr; ^3^ Medicinal and Natural Products Chemistry Research Center, Shiraz University of Medical Sciences, Shiraz, Iran, sums.ac.ir; ^4^ Plant and Animal Production Department, Technical Sciences Vocational School of Sivas, Sivas Cumhuriyet University, Sivas, Turkey, cumhuriyet.edu.tr

**Keywords:** anticancer, antiepileptic, antioxidant, enzyme inhibition, molecular docking

## Abstract

**Introduction:**

This study aimed to investigate the anticancer, potential antiepileptic agents, and antioxidant potentials of 10 methyl‐substituted halogenated and methoxy conduritols, which had been previously synthesized and characterized. The presence of active functional groups within their structures suggested their potential as bioactive molecules. Both in vitro and in silico approaches were employed to assess their biological activities and therapeutic relevance.

**Methods:**

Anticancer activity was tested using the MCF‐7 breast cancer cell line and the L929 fibroblast cell line, with IC_50_ values calculated to evaluate cytotoxicity. Antioxidant activity was determined using DPPH, FRAP, and TAS assays. The effects of methyl‐substituted mono‐ and dimethoxy halogenated conduritol derivatives (A and B forms) on the activities of carbonic anhydrase isoenzymes hCA I and hCA II were examined spectrophotometrically. Additionally, molecular docking studies were performed against hCA I (PDB ID: 3LXE), hCA II (PDB ID: 5AML), and breast cancer proteins (PDB ID: 1JNX, 1A52). ADME/T properties of the compounds were also evaluated to predict their pharmacokinetic and safety profiles.

**Results:**

Among the synthesized derivatives, only compound 6 demonstrated notable anticancer activity, with an IC₅₀ of 20.22 *μ*M against MCF‐7 cells and moderate selectivity over healthy fibroblasts. The other compounds were largely inactive at the tested concentrations. Antioxidant assays demonstrated considerable free radical scavenging and reducing power. The synthesized conduritols showed strong inhibition of carbonic anhydrase isoenzymes, with Ki values ranging from 0.2083 ± 0.11 to 1.4944 ± 1.06 *μ*M for hCA I and 0.0857 ± 0.06 to 2.2098 ± 0.68 *μ*M for hCA II, outperforming standard inhibitors. Docking studies confirmed strong binding affinities to the investigated proteins, while ADME/T analysis suggested favorable pharmacokinetic properties.

**Conclusions:**

The findings indicate that methyl‐substituted halogenated and methoxy conduritols possess anticancer, potential antiepileptic agents, and antioxidant potentials. Their strong carbonic anhydrase inhibitory activities highlight their promise as potential therapeutic agents for epilepsy and glaucoma. Overall, these compounds demonstrate considerable potential as multifunctional bioactive molecules and represent promising candidates for further preclinical studies.

## 1. Introduction

**Figure 1:**
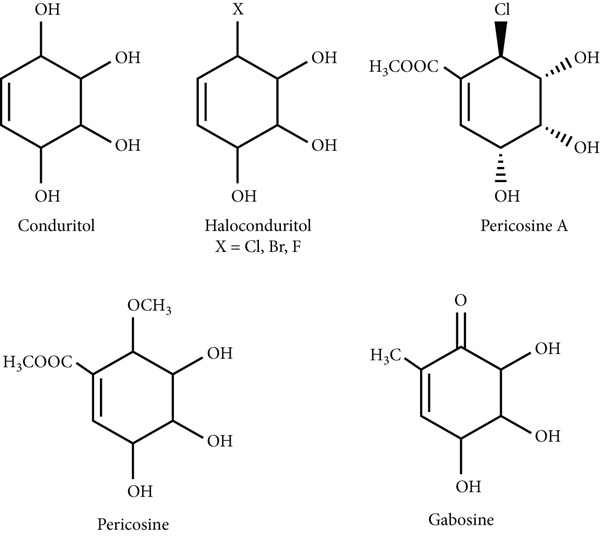
Compounds containing selected ▬OH groups.

Conduritols are compounds similar to carbasugars and find application in various biological processes [1–3]. In addition, their derivatives, benzo‐ [4–6], methyl‐ [7], and halo‐conduritols [8], have attracted the attention of many researchers. A number of conduritol derivatives have been found to possess antifeedant, antibiotic, antileukemic, and growth‐regulating activity [9]. On the other hand, pericosins and gabosins containing methoxy and halogen functional groups are from the carbasugar class and are important biologically active compounds in Figure 1 [10–14].

Carbonic anhydrase (CA; carbonate hydrolyase, EC 4.2.1.1) is a metalloenzyme that functions in a wide variety of tissues [15–21]. It is a catalyst that facilitates the reversible hydration of carbon dioxide to bicarbonate for the purpose of producing bicarbonate. This process is facilitated by the presence of zinc (Zn^2+^) ions in its active site, which play the role of a catalyst. The majority of the compounds that are classified as CA inhibitors (CAIs) can be derived from sulfonamide. These CAIs have either been approved by the Food and Drug Administration (FDA) or are currently being developed through clinical trials. It has been demonstrated that one of the factors that contributes to off‐target hCA inhibition is the lack of specificity that is present in the compounds that are currently available. Because of this inhibition, a wide variety of unfavorable side effects are brought about, ranging from feelings of nausea and fatigue to feelings of psychological depression. Among the 16 human isoforms, all of them have sequence and structural similarities, particularly within the active site. These similarities are particularly prevalent. This makes the process of designing inhibitors that are specific to isoforms a challenging endeavor. Because of this, structure‐based drug design techniques look for residues that are unique to that isoform and make use of them in their design process. It is the HCAs in the brain that are accountable for the active regulation of pH buffering in both the extracellular and intracellular spaces. Catalyzing the reversible hydration of carbon dioxide is the means by which they accomplish this. Seizure activity is therefore regulated and modulated by hCAs, possibly through the maintenance of brain pH and CO_2_ levels. The hCA is considered an interesting target for the development of potent anticonvulsant agents, and its inhibition provides antiepileptic activity. This is because some of the antiepileptic drugs that are currently being used in clinical settings exhibit potent hCA inhibitory activity. The fact that hCA inhibitors are not isoform‐specific, on the other hand, results in a lack of specificity and, consequently, a variety of adverse effects. In order to reduce the severity of these adverse effects and to find new medications that have superior pharmacological properties to those of the currently available medications, there is a need for additional research on chemicals that have the potential to treat epilepsy.

With roughly 3%–6% of the general population thought to be allergic to sulfonamides, synthetic derivatives of p‐aminobenzenesulfonamide, there is also a population that cannot be treated by sulfonamides because they are allergic to sulfur‐based substances. For all of these reasons, there is an increased attempt to create nonclassical CAIs that are isoform‐specific [22–30].

Breast cancer is a type of cancer whose incidence is increasing and its treatment has not been fully explored yet. Anticancer drugs used in today’s treatment also damage other healthy tissues due to their toxic properties. For this reason, the discovery of more specific inhibitory compounds with targeted, new nanotechnological methods and reduced side effects in breast cancer is increasing rapidly. The most commonly used MCF‐7 human breast cancer cell line in breast cancer is Luminal A type tumors that make up 40%–70% of all breast cancers and have the best prognosis because of their slow growth [31]. Anticancer drugs, which are used in breast cancer as well as in cancer treatment, cause cell death by creating DNA damage. As a result, DNA repair pathways that create drug resistance come into play. Considering the resistance developed by the body against the drug, many new alternative drugs should be added to the cancer drugs available in the market.

Conduritols are molecules with important biological activity and form the building blocks of many natural products. Since such molecules generally contain many hydroxyl (▬OH) functional groups, they are found in the structure of many pharmacological drugs [3, 32]. In addition, halogenated and methylated derivatives of such compounds are also important compounds [2, 33, 34]. Therefore, it is a focus of intense interest in the medical field as well as synthetic chemists [5]. It is thought that conduritol derivatives targeted for synthesis will have the characteristics to show biological activity. As a result, such new compounds may find use in further activity studies.

The use of theoretical computations is a common method that is employed in the process of comparing the activities of molecules. In order to design molecules that are more efficient, it is necessary to perform theoretical calculations that pertain to the interactions that occur between molecules and proteins. More specifically, the calculations concentrate on the active sites of molecules [35, 36].

Antiepileptic medications currently on the market are unable to fully address the seizure issue in patients with epilepsy. New antiepileptic chemicals that differ structurally and pharmacologically from the currently recommended medications are required since the most widely used CAIs, acetazolamide and methazolamide, do not offer long‐term effective treatment and quickly induce tolerance. Furthermore, sulfonamides, which are synthetic derivatives of *p*‐aminobenzenesulfonamide, are thought to cause allergies in 3%–6% of the general population. As a result, sulfonamide‐structured CAIs are not suitable for these patients. Current medications have a number of adverse effects since they are unable to selectively inhibit CA isozymes. Because of all these factors, our research helps to create several approaches for designing more selective CAIs as antiepileptic medications.

This study will involve the synthesis of methyl‐substituted mono‐ and dimethoxy conduritols. Inhibition studies of CA enzyme for the synthesized compounds were conducted, and IC_50_ and Ki values were determined. The antioxidant capacity of these molecules was assessed. Ultimately, the anticancer study will calculate IC_50_ values to assess the efficacy of the substances on the MCF‐7 human breast cancer cell line, and the characteristics of these identified compounds as alternative therapeutics will be examined. To clarify the biological structures of these compounds, initial in vitro studies were conducted on new compounds exhibiting high biocompatibility as alternatives for epilepsy, glaucoma, neurodegenerative diseases, diuretics, and breast cancer medications. The proteins employed for comparing the activities of the molecules are hCA I (PDB ID: 3LXE) [37], hCA II (PDB ID: 5AML) [38], and breast cancer proteins (PDB IDs: 1JNX and 1A52) [39, 40]. Subsequently, ADME/T calculations were conducted to forecast the behavior of molecules in human metabolism.

## 2. Materials and Methods

### 2.1. Chemistry

**Figure 2:**
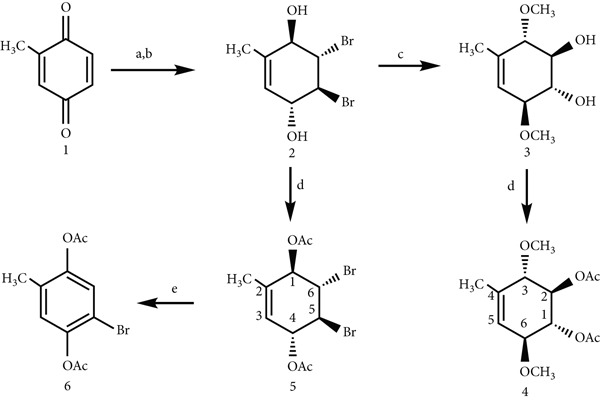
Synthesis of compounds **2-5** and **6;** Reagents and conditions: (a) Br_2_, CH_2_Cl_2_, −5°C; (b) NaBH_4_, Et_2_O/H_2_O, −15°C, 85%; (c) CH_3_ONa/CH_3_OH, 0°C, N_2_(g), 76%; (d) Ac_2_O/pyridine, 0°C, N_2_(g), 89%, (for **5**, 86%); (e) AcOK/AcOK, Ac_2_O, reflux, 80%.

The allylic *trans*‐diol **2** is obtained, according to the literature, by bromination of methyl‐substituted para‐benzoquinone followed by reduction of carbonyl groups with sodium borohydride. Conduritol‐B derivative **3** [7] was obtained stereo‐controlled from the reaction of dibromodiol **2** with the metallic sodium–methanol system (MeONa/MeOH) [4] and subsequent treatment of the hydroxyl groups with the Ac_2_O/pyridine system gave dimethoxy‐diacetoxy conduritol B derivative **4** (89% yield). Compound **5** was obtained by acetylation of compound **2**, while treatment of compound **5** with AcOK and Ac_2_O in acetic acid yielded aromatic compound **6** in high yield in Figure 2 [7].

LiOH was used as the base in the regioselective preparation of epoxide **7**, which was derived from dibromide **5** [7]. Through a two‐step process, bromotriacetate **9**, which is a derivative of conduritol A, was produced from epoxide (see Supporting Information) [7]. When the acetate functionalities in compounds **9** were removed using NaOMe in methanol, the methyl‐substituted methoxy conduritol **10** that was anticipated was produced in virtually quantifiable yield (93% yield) (Scheme 2) [7]. In contrast, the regio‐ and stereoselective ring opening of epoxide **7** with AcCl led to the formation of dihalo‐diacetate compound **11** (56% yield), which is the sole reaction product shown in Figure 3 [8]. This dihalo‐diacetate has a conduritol‐A‐type conformation. Detailed synthetic procedures and additional characterization data are provided in the Supplementary Data and Figures S1–S23.

**Figure 3:**
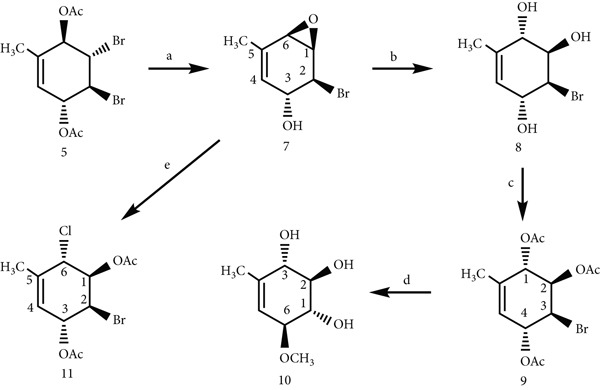
Synthesis of compounds 7–11; Reagents and conditions: (a) LiOH, Et_2_O/H_2_O, 0°C, 70%; (b) CBr_4_, H_2_O, 35°C, 51%; (c) Ac_2_O/pyridine, rt, 78%; (d) CH_3_ONa, 5°C, N_2_(g), 93%; (e) AcCl/CH_2_Cl_2_, 0°C to rt, 56%.

### 2.2. Enzyme Studies

An esterase activity technique was utilized in order to determine the level of enzyme activity exhibited by CA. This method is based on the esterase activity of CA as its building block. The utilization of nitrophenyl acetatenylasetate as a substrate by the enzyme is the concept that underpins the CA catalyst. The hydrolysis process results in the production of either *p-*nitrophenol or *p*‐nitrophenol, which absorbs light at a wavelength of 348 nm [41].

Using this method, the absorbance characteristics of *p*‐nitrophenol and *p*‐nitrophenolate are identical at a wavelength of 348 nm. A consequence of this is that the generation of phenol or phenolate does not have any impact on the measurement that takes place during the reaction. As a result of the fact that 348 nm *p*‐nitro phenyl acetate offers very little absorption, it is utilized in a blind manner. During the measurements, an activity determination strategy was utilized, and the application of it is accomplished by combining the reaction mixture in quartz cuvettes of 3 mL.

### 2.3. Antioxidant Activity

There have been a lot of different approaches developed to figure out antioxidant capacity. In accordance with the radicals or the target molecule, these methods are distinct from one another. When it comes to determining antioxidant capacity, there is no one method that can completely evaluate antioxidant capacity. For this reason, in subsequent research, an attempt will be made to ascertain the antioxidant capacity by employing a variety of different techniques. Methods such as DPPH, ABTS, CUPRAC, FRAP, and FCR are examples [42] within the category of methods that can be used to determine the antioxidant capacity of a substance.

#### 2.3.1. Activity of the TAS

A commercial kit, which was described in a previous article [42], is used to determine the total antioxidant status (TAS) of conduritol derivatives. The Trolox equivalent, which is the vitamin E analogue, was used to calibrate the assay. Immediately following the implementation of the kit method that was mentioned, calculations were carried out, and the outcomes were reported.

#### 2.3.2. DPPH

Sánchez‐Moreno et al. [43], who were the ones responsible for its development and utilization, utilized the DPPH method, which was initially developed by Brand‐Williams et al. in the year 1995. Sánchez‐Moreno et al. [43] were the ones who were responsible for its incorporation. A stable organic radical that can be purchased in the market is referred to as DPPH, which is an abbreviation that stands for 2,2‐diphenyl‐1‐picrylhydrazil [44]. It is also possible to use this analysis to measure the antioxidant capacity of structures that have been synthesized [45]. This analysis is used to measure the antioxidant capacity of natural extracts on a regular basis. Naturally occurring extracts are subjected to the DPPH radical scavenging capacity analysis in order to determine their level of antioxidant capacity. This method is distinguished by a number of fundamental characteristics, the most fundamental of which is the change in absorbance at 517 nm that occurs as a result of the transfer of a proton to the DPPH free radical by the potential antioxidant [46]. With regard to the methanol solution that is being taken into consideration, it has been discovered that the DPPH radical has the highest absorbance at a wavelength of 517 nuclear meters. The fact that this method is considered to be both straightforward and speedy is the reason why it is utilized in a significant number of research projects. However, the method has a number of drawbacks, such as the fact that it is susceptible to oxygen, light, and pollution. These are just some of the drawbacks.

#### 2.3.3. FRAP

Benzei and Strain are the ones who came up with the FRAP method, which is a technique that utilizes antioxidants in order to determine the reducing capacity of iron (III). They are the ones who conceived of the method in the first place. The formation of a complex that is referred to as Fe (III)‐TPTZ is the end result of the reaction that takes place between tripyridyltriazine (TPTZ) and iron (III). In the subsequent step, the complex is reduced to the Fe (II)‐TPTZ complex by making use of the antioxidant that is present in the environment that is surrounding it. Specifically, this particular complex reaches its maximum absorbance at a wavelength of 593 nm [47], and it also has a color that is described as being dark blue. In order to acquire accurate absorbance values, the reaction is allowed to proceed for a period of up to 30 min while the incubation process is being carried out. This is done in order to ensure that the absorbance values are accurate. The results that were obtained can be described using either the Trolox equivalent or the IC_50_ values [46]. Both of these methods are viable options.

### 2.4. Anticancer Effect

The absorbance values that were obtained from MTT assays were utilized for the purpose of determining the anticancer activities of any conduritol derivatives that were present. After careful consideration, it was decided that the MTT method ought to be carried out in accordance with the description that came before it [42]. These compounds were put through a series of tests against the MCF‐7 breast cancer cell line in order to ascertain whether or not they possess the capability to inhibit the growth of cancer cells. The test subjects consisted of L929 healthy mouse fibroblast cells, and the purpose of the experiment was to determine the selectivity of the compounds. Within the scope of this particular section of the manuscript, cisplatin was employed as a reference drug in cell lines.

#### 2.4.1. Annexin V Binding Assay

After being seeded into six‐well plates at a density of approximately 5 × 10^5^, the seeds of each cancer cell were allowed to adhere for a full 24 h before being removed. Compound 6, which was then incubated at the IC_50_ dose for an additional 24 h, was concluded to be the most effective compound for MCF7 cancer cells. This conclusion was reached after the compound was tested. After trypsinization, the cells were harvested and then suspended in PBS that contained at least 1% FBS. This process was repeated until the cells were completely harvested. Following that, the Annexin V & Dead Cell reagent was mixed with the cells, and the instructions that were provided by the manufacturer were followed with great thoroughness. In the subsequent step, the Muse Cell Analyzer, which is manufactured by Millipore, was utilized in order to determine the percentage of cells that were either dead, viable, early, or late in the apoptotic process.

#### 2.4.2. Cell Cycle Assay

The MUSE flow cytometry device was utilized in order to carry out DNA content (cell cycle) analyses. Following the evaluation of the IC_50_ results that were obtained, the cancer cell line MCF7 was resistant to the effects of compound 6, which caused selective toxicity in comparison to L929, respectively. As a result, the MUSE (cell cycle) kit was utilized in order to ascertain the primary pathways that compound 6 takes within cells.

### 2.5. Theoretical Calculation

The molecular docking approach is one of the ways that is used for the purpose of determining which compounds have the greatest activity against biological materials. This is an essential method that is used to identify which molecules have the highest activity vs. biological materials. In order to carry out calculations for the purpose of molecular docking, Schrodinger’s Maestro Molecular modeling platform (version 12.8) [48] is utilized. Because of the calculations that are performed using this approach, it is feasible to make observations about the active sites that are present in the molecules. These observations may be made concerning the active sites. There are a variety of stages involved in the process of performing calculations. Following the preparation of the protein, which is accomplished through the utilization of the protein preparation module [49], it then proceeds to the preparation of the molecules through the utilization of the LigPrep module [50]. Using the Glide ligand docking tool [51, 52], the proteins and molecules that have been produced connect with one another in order to determine the way in which they will interact with one another in the future. This is done in order to determine how they will interact with one another. In conclusion, the Qik‐prop module of the Schrodinger software [53] was employed in the process of doing ADME/T analysis (absorption, distribution, metabolism, excretion, and toxicity) in order to explore the effects and effects of the chemicals that were researched on human metabolism. This was done in order to determine the impacts that the molecules had on different aspects of human metabolism. For the purpose of determining the impact that the compounds had on various elements of human metabolism, this was carried out.

## 3. Results

### 3.1. CAs Inhibition Activity Results

To examine the inhibitory potentials of methyl substituted mono‐ and dimethoxy conduritol derivatives against two physiologically relevant CA isoforms, the esterase assay technique was applied. This was done in order to determine the potentials of these synthetic compounds. Both of these isoforms are referred to as cytosolic isoenzyme (hCA II) and cytosolic isoform (hCA I), with the latter being the more rapid of the two. In Table 1, which contains IC_50_ and Ki values presented in micromolar units, you will find a summary of the compound‐by‐hCA I and II isoform inhibition data. This table also provides additional information.

**Table 1:** The enzyme inhibition results of novel compounds against carbonic anhydrase I and II isoenzymes.

**Comp.**	**IC_50_ (*μ*M)**				**K_i_ (*μ*M)**	
	**HCA I**	**r^2^**	**HCA II**	**r^2^**	**HCA I**	**HCA II**
2	0.937	0.963	0.581	0.933	1.17 ± 0.50	2.21 ± 0.68
3	1.197	0.943	0.651	0.983	1.42 ± 0.57	1.49 ± 1.16
4	0.932	0.975	0.362	0.978	0.34 ± 0.08	0.53 ± 0.22
5	1.167	0.864	0.331	0.957	0.98 ± 0.22	0.61 ± 0.26
6	0.407	0.929	1.038	0.941	0.43 ± 0.18	0.71 ± 0.05
7	1.106	0.966	0.595	0.957	1.36 ± 0.47	0.08 ± 0.06
8	0.540	0.927	0.404	0.950	0.20 ± 0.11	0.24 ± 0.02
9	0.911	0.900	0.353	0.936	1.49 ± 1.06	0.56 ± 0.16
10	1.439	0.963	0.574	0.932	1.40 ± 0.26	0.81 ± 0.41
11	2.944	0.980	0.627	0.933	0.81 ± 0.08	0.69 ± 0.13
AZA^a^	18.110	0.938	20.650	0.975	22.35 ± 3.63	26.52 ± 6.06

^a^AZA (acetazolamide) was used as a positive control for human carbonic anhydrase I and II isoforms (hCA I and II).

**Figure 4:**
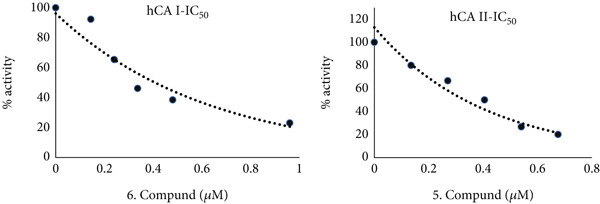
IC_50_ graphs for molecules with the most effective inhibition values against carbonic anhydrase I and II isoenzymes.

**Figure 5:**
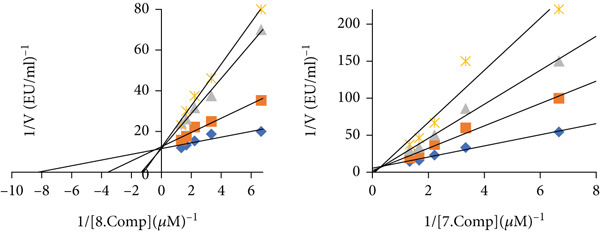
K_İ_ graphs for molecules with the most effective inhibition values against carbonic anhydrase I and II isoenzymes.

**Figure 6:**
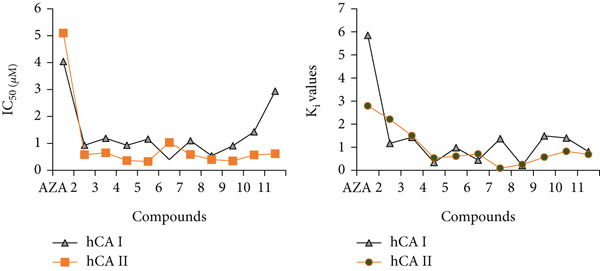
Graphs showing the change in IC_50_ and K_i_ values for all molecules against carbonic anhydrase I and II isoenzymes.

All of the methyl substituted mono‐ and dimethoxy conduritol compounds displayed a strong suppression of both the cytosolic isoforms of hCA I (with an IC_50_ falling somewhere between 0.40 and 2.94 *μ*M) and hCA II (with an IC_50_ falling anywhere between 0.33 and 1.08 *μ*M). The most effective inhibitor for human chorionic acid (hCA I) was found to be compound 6, whereas compound 5 was found to be the most effective inhibitor for human chorionic acid (hCA II). Both of these compounds were shown to be efficient in inhibiting human chorionic acid. According to Figures 4 and 5, the IC_50_ values for these two compounds were 0.40 and 0.33 *μ*M, respectively. These values may be seen in the corresponding figures. Both human choroidal adenosine monophosphate (hCA I) and human choroidal adenosine II (hCA II) are demonstrated to be inhibited by methyl substituted mono‐ and dimethoxy conduritols derivatives (2‐11). These inhibitory effects are shown in Table 1 and Figure 6, respectively. These substances have Ki values that range from 0.08 ± 0.06–2.21 ± 0.68 *μ*M, which is a wide range.

When the data that were obtained were compared with the acetazolamide that was utilized as a standard, it was discovered that the IC_50_ and Ki values were much greater than those of the standard material. When the findings were compared with the standard material, this was shown to be the case. When it comes to their respective values, the IC_50_ and Ki values are quite complementary to one another.

Patients who are suffering from a broad variety of disorders, including glaucoma, epilepsy, edema, and ocular hypertension, may be treated with hCA inhibitors and get treatment for their condition. It is beneficial in the treatment of a wide range of illnesses, provided that it is delivered in the appropriate manner. Alternative medicine is always doing research in order to develop medications that have a low risk of harmful effects while yet having a high potential for pharmacological advantages. This is done in order to promote the field of alternative medicine. The purpose of this endeavor is to locate drugs that are both of these things, and this is something that is being done. There are a number of neurological conditions, such as epilepsy and Alzheimer’s disease, that cannot be completely treated with the medications that are now available from the pharmaceutical industry. As a direct result of this, there is an urgent need for medications that are more effective in their intended purpose and have a limited range of adverse effects. According to the data obtained, these compounds may represent an alternative class of CAIs, distinct from classical sulfonamide‐based scaffolds. This perception is supported by our inhibition and docking results, and is consistent with previous reports describing nonclassical CAIs such as phenols and coumarins that interact with isoform‐specific residues outside the zinc‐binding site [28, 54]. In addition, recent studies have shown that structural modifications of small molecules can yield potent hCA I/II inhibitors with anticancer potential, further supporting our findings [55, 56].

### 3.2. Antioxidant Activity

#### 3.2.1. TAS

In order for the total antioxidant capacity level to be defined as high or desired level, it is expected to have a value equal to or greater than 1.0 mmol Trolox Equiv./L. The values of the studied syntheses are shown in Table 2. Although compounds 2 and 6 show high antioxidant capacity values, compound 10 showed highest value even higher than Vitamin E itself.

**Table 2:** mmol Trolox Equiv./L for each compound.

Vitamin E	1.000 ± 0.097
2	0.728 ± 0.011
3	0.258 ± 0.004
4	0.182 ± 0.046
5	0.219 ± 0.003
6	0.858 ± 0.081
7	0.243 ± 0.034
8	0.349 ± 0.018
9	0.438 ± 0.010
10	1.256 ± 0.086
11	0.143 ± 0.056

#### 3.2.2. DPPH

In order to complete the objective of establishing the total free radical scavenging capacity of each sample, a series of solutions ranging from 50 to 1000 *μ*M were generated and compared with vitamin C using stable DPPH, which is made previously [57]. This was done in order to accomplish the mission. Within the framework of the technique that Yu et al. [58] had previously used, this comparison was carried out in line with that methodology. At a ratio of one to one, a newly made 0.1 mM DPPH solution with ethanol was added to all of the different concentrations of samples that were created, as well as to the various concentrations of vitamin C solutions that were used as a standard. This was done in order to maintain consistency throughout the experiment. At the same time as the DPPH solution was added to the ethanol solution in order to act as a negative control, the measurement was also carried out in this way. After adding all of the solutions to the microplates, the plates were then put in a dark setting and incubated for a period of 60 min without any light sources. In order to determine the UV absorbance values at a wavelength of 517 nm, a spectrophotometer known as the BMG LABTECH SPECTROstar Nano was used. The temperature was maintained at room temperature during the experiments. The findings of this research are reported as the mean plus or minus the standard deviation (*n* = 3), and they are compared with the naturally occurring antioxidant ascorbic acid. A total of three participants participated in this investigation. The IC_50_ values were determined by comparing the radical scavenging activity of the samples and standards to the activity of the negative control. This was done in order to determine the IC_50_ values in Table 3. During the course of the investigation, it was discovered that compounds 3, 4, 5, 7, 8, 9, and 11 demonstrated scavenging characteristics for DPPH that were lower than those of ascorbic acid. This was the conclusion reached by the researchers. On the other hand, it was discovered that compounds 2, 6, and 10 each exhibited a larger potential for scavenging than ascorbic acid did.

**Table 3:** IC_50_ values of ascorbic acid and other compounds against DPPH as *μ*M.

Ascorbic acid	227.08 ± 0.186
2	213.71 ± 0.022
3	406.40 ± 0.005
4	573.22 ± 0.013
5	516.96 ± 0.021
6	183.20 ± 0.028
7	482.48 ± 0.019
8	590.37 ± 0.003
9	863.78 ± 0.010
10	174.56 ± 0.001
11	974.29 ± 0.002

#### 3.2.3. FRAP

In the same way as some reducing agents do, antioxidants are responsible for the reduction of the Fe^+3^ ferricyanide complex to only Fe^+2^. Because of the fact that the reducing power of the material being tested is taken into consideration, the color of the test solution shifts from yellow to green when using this approach. This green hue has the highest absorbance at 700 nm, and if the absorbance increases, it implies that the reduction strength is also growing. According to this approach, trolox was used as the standard antioxidant component, and measurements were carried out in line with the protocol that was established by Benzie and Strain [58]. It was found, based on the IC_50_ values that were acquired as a consequence of the investigation, that the compounds 2 and 10 exhibited stronger antioxidant characteristics than vitamin E for iron reduction. These findings are shown in Table 4.

**Table 4:** IC_50_ values of vitamin E and other compounds against reducing for Fe^+3^ as *μ*M.

Vitamin E	0.629 ± 0.017
2	0.481 ± 0.013
3	0.823 ± 0.069
4	0.915 ± 0.038
5	0.767 ± 0.072
6	0.761 ± 0.009
7	1.299 ± 0.073
8	1.336 ± 0.075
9	0.955 ± 0.041
10	0.325 ± 0.013
11	1.953 ± 0.041

### 3.3. Anticancer Effect

**Figure 7:**
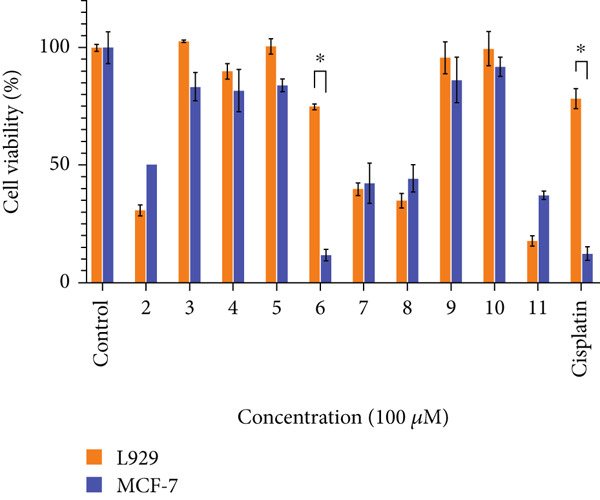
Cell viability for MCF‐7 and L929 cell lines at 100 *μ*M synthesis concentration (∗*p* < 0.05).

Anticancer effect of compounds 2–11 was evaluated against MCF‐7 cell line. Cytotoxic bioactivities of L929 and MCF‐7 cell lines were determined by MTT assay, which is explained previously [59–62]. As a result of the experiment, in which each cell was exposed to a constant concentration of 100 *μ*M, the absorbance values were measured after 48 h. Cell viability was calculated with the absorbance values found. Preliminary anticancer effect results of compounds 2–11 against L929 and MCF‐7 are presented in Figure 7. The MTT study was performed in triplicate for each synthesis. After observing the effects of maximum doses on cell viability, MTT studies were performed in triplicate at different concentrations (100, 50, 25, 12, 6, and 3 *μ*M, respectively) to calculate IC_50_ doses. The majority of compounds (2–11) did not exhibit significant anticancer activity under the tested conditions. However, **compound 6** stood out with selective cytotoxicity in MCF‐7 cells, while sparing normal fibroblasts at equivalent doses which shown in Table 5.

**Table 5:** IC_50_ values of each synthesis for MCF‐7 and L929 cell lines.

	**IC_50_ values (*μ*M)**	
	**L929**	**MCF-7**
2	72.2 ± 1.73	> 100
3	> 100	> 100
4	> 100	> 100
5	> 100	> 100
6	88.77 ± 0.71	20.22 ± 1.97
7	82.96 ± 2.27	86.65 ± 7.44
8	76.67 ± 2.32	89.7 ± 5.2
9	> 100	> 100
10	> 100	> 100
11	60.78 ± 1.37	79.51 ± 1.42
Cisplatin	> 100	21.49 ± 1.97

#### 3.3.1. Flow Cytometry

**Figure 8:**
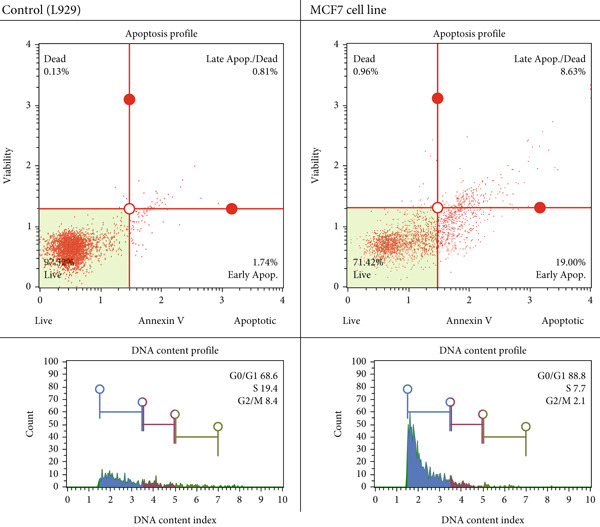
After 24 h of incubation apoptotic and cell cycle consequences of compound 6 on L929 and MCF7 with IC_50_ dose (20.22 ± 1.97 *μ*M).

Using the flow cytometry tests that were reported earlier [63], the most effective therapy on the MCF7 cell line was listed as compound 6. Additionally, the manner in which it was guided to apoptosis using Annexin V dye was detailed, as well as the location where the cell cycle was halted using the required kit. The outcomes that were brought about are shown in Figure 8.

The proportion of live cells for compound 6 was 97.32% when the IC_50_ dosage was administered to the healthy cell line. However, 2.55% of the cells passed through the apoptosis stage for compound 6. The proportion of viable cells was found to be 71.42% in the flow cytometry research that was carried out in breast cancer cell line at the same dosage for compound 6. On the other hand, the percentage of cells that progressed to apoptosis was found to be 27.63%. A cyclic disruption was not found in healthy cells during the cell cycle analysis that was carried out by using the same IC_50_ value. However, an accumulation of 88.8 percent was observed in the G0/G1 phase of the MCF7 cell line for compound 6.

### 3.4. Theoretical Calculations

**Figure 9:**
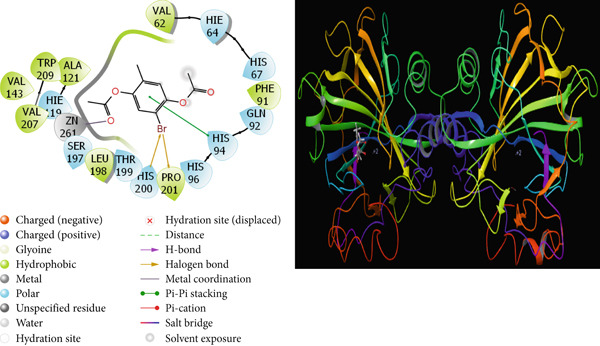
Presentation interactions of molecule **6** with hCA I enzyme.

**Figure 10:**
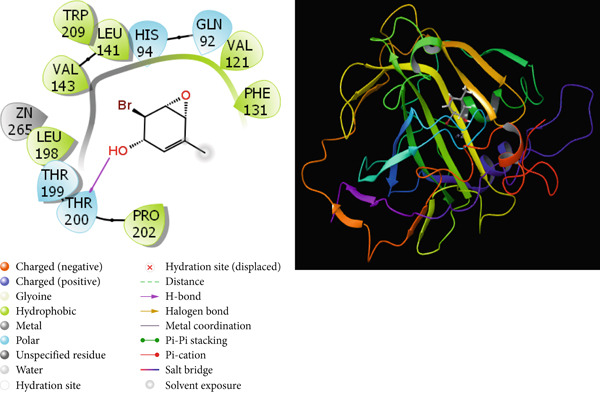
Presentation interactions of molecule **7** with hCA II enzyme.

**Figure 11:**
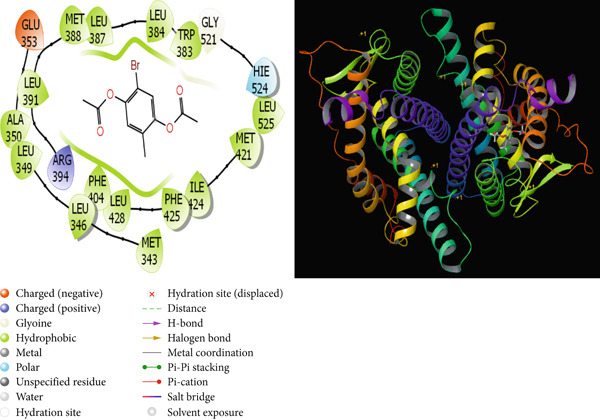
Presentation interactions of molecule **6** with breast cancer protein (1A52).

**Figure 12:**
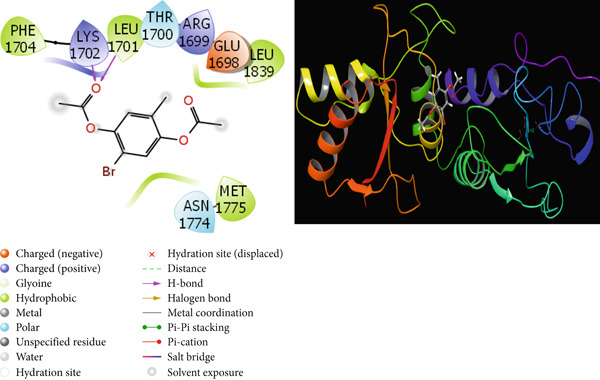
Presentation interactions of molecule **6** with breast cancer protein (1JNX).

It is one of the important methods used to predict the activities of molecules and to design effective drugs by examining the interactions that occur between molecules and proteins with theoretical calculations [64, 65]. Molecular docking calculations are an important theoretical method used to design new and effective molecules by finding the interaction sites of molecules. A cure is sought for this disease by enabling molecules to inhibit various disease proteins. In the calculations, it is thought that molecules that interact more with these proteins inhibit these proteins. It is known that the activity of the molecule that inhibits more is higher than the other molecules. These interactions are hydrogen bonds, polar and hydrophobic interactions, *π*‐*π* and halogen [66, 67]. The interactions between the molecules with the highest activity and the proteins are given in Figures 9, 10, 11, and 12.

As a result of the calculations, there are many parameters. These are given in Table 6. Among these parameters, the docking score parameter is the parameter used to comment on the activities of the molecules. Parameters such as Glide ligand efficiency, Glide hbond, Glide evdw, and Glide ecoul are the parameters that give the numerical value of the interactions between molecules and proteins [68]. Parameters such as Glide emodel, Glide energy, Glide einternal, and Glide posenum are the parameters of numerical values about the interaction pose that occurs between molecules and proteins [69].

**Table 6:** Numerical values of the docking parameters of molecules against some proteins.

**hCA I**	**Docking score**	**Glide ligand efficiency**	**Glide hbond**	**Glide evdw**	**Glide ecoul**	**Glide emodel**	**Glide energy**	**Glide einternal**	**Glide posenum**
2	−5.62	−0.51	−0.61	−10.23	−16.42	−37.55	−26.65	2.20	311
3	−5.23	−0.44	−0.24	−22.56	−7.97	−39.94	−30.53	2.92	278
4	−5.94	−0.33	−0.70	−28.83	−9.32	−53.21	−38.16	5.39	351
5	−5.20	−0.31	−0.30	−31.68	−7.61	−52.74	−39.29	1.28	300
6	−5.95	−0.22	−0.02	−23.38	−5.44	−35.95	−28.82	2.89	146
7	−5.48	−0.55	−0.32	−13.64	−10.41	−33.51	−24.05	0.91	213
8	−5.94	−0.54	−0.56	−15.98	−11.33	−38.44	−27.31	0.92	361
9	−5.42	−0.27	−0.56	−34.23	−5.74	−51.43	−39.97	5.80	154
10	−5.89	−0.49	−0.28	−13.79	−10.12	−34.91	−23.91	2.43	201
11	−5.33	−0.31	−0.61	−27.54	−8.44	−48.27	−35.98	3.87	75
**hCA II**	**Docking score**	**Glide ligand efficiency**	**Glide hbond**	**Glide evdw**	**Glide ecoul**	**Glide emodel**	**Glide energy**	**Glide einternal**	**Glide posenum**
2	−5.30	−0.48	−0.52	−17.67	−12.14	−38.04	−29.81	1.46	262
3	−5.20	−0.43	−0.61	−13.08	−13.67	−34.37	−26.76	1.91	359
4	−5.65	−0.31	−0.62	−24.27	−12.72	−48.83	−36.99	5.97	245
5	−4.90	−0.29	−0.46	−29.38	−9.79	−51.57	−39.17	1.31	381
6	−4.13	−0.26	−0.39	−25.54	−6.77	−39.95	−32.31	4.78	116
7	−6.42	−0.54	−0.32	−14.27	−11.42	−34.24	−25.69	0.76	20
8	−5.86	−0.53	−0.24	−12.67	−17.34	−38.82	−30.01	3.34	391
9	−4.63	−0.23	−0.20	−26.09	−10.11	−46.18	−36.20	3.37	74
10	−6.28	−0.52	−0.04	−2.51	−21.72	−32.19	−24.23	5.11	263
11	−5.37	−0.32	−0.48	−27.01	−9.09	−45.22	−36.10	5.99	372
1JNX	**Docking score**	**Glide ligand efficiency**	**Glide hbond**	**Glide evdw**	**Glide ecoul**	**Glide emodel**	**Glide energy**	**Glide einternal**	**Glide posenum**
2	−4.78	−0.43	−0.61	−12.80	−10.62	−28.18	−23.43	3.10	110
3	−4.17	−0.35	−0.53	−15.16	−6.21	−25.71	−21.37	2.99	266
4	−4.11	−0.23	−0.30	−13.59	−9.53	−28.18	−23.12	3.70	63
5	−3.43	−0.20	−0.33	−19.59	−5.54	−30.03	−25.13	1.14	3
6	−5.85	−0.19	0.00	−20.95	−3.98	−29.29	−24.93	0.86	247
7	−4.75	−0.48	−0.41	−10.53	−7.53	−23.21	−18.06	0.63	378
8	−5.46	−0.50	0.00	3.82	−25.08	−30.66	−21.26	2.95	224
9	−3.60	−0.18	−0.51	−20.41	−7.12	−33.98	−27.54	1.29	194
10	−5.09	−0.42	−0.12	−8.46	−13.41	−27.77	−21.87	5.23	166
11	−3.64	−0.21	−0.37	−19.92	−5.13	−30.44	−25.05	1.00	302
1A52	**Docking score**	**Glide ligand efficiency**	**Glide hbond**	**Glide evdw**	**Glide ecoul**	**Glide emodel**	**Glide energy**	**Glide einternal**	**Glide posenum**
2	−6.08	−0.55	−0.28	−23.00	−2.75	−33.79	−25.75	2.44	176
3	−6.33	−0.53	−0.20	−25.69	−2.47	−37.18	−28.16	2.43	181
4	−6.88	−0.38	0.00	−33.19	−2.74	−47.12	−35.93	6.09	193
5	−6.61	−0.39	0.00	−34.93	−2.30	−48.49	−37.23	7.83	56
6	−7.65	−0.35	0.00	−29.02	−1.86	−41.81	−30.88	1.22	161
7	−6.41	−0.64	−0.32	−19.28	−2.44	−30.45	−21.72	0.42	9
8	−6.10	−0.55	−0.32	−21.35	−2.58	−32.67	−23.93	0.46	198
9	−6.20	−0.31	0.00	−36.20	−0.11	−44.34	−36.32	9.52	40
10	−6.13	−0.51	0.00	−17.13	−6.78	−31.59	−23.91	2.84	334
11	−7.11	−0.42	−0.20	−35.04	−0.83	−48.70	−35.87	4.29	96

As a result of the calculations, when the interactions of the studied molecules with various proteins are examined, when the interaction of molecule 6 with the hCA I protein is examined in Figure 9, it is seen that the benzene ring in the center of molecule **6** makes a Pi‐Pi stacking interaction with the HIS 94 protein. However, the bromine atom attached to this benzene ring appears to form a halogen bond with the proteins HIs 200 and PRO 201. It is seen that the oxygen atom attached to the carbonyl carbon in molecule **6** makes metal interaction with the Zn 261 metal atom. The interaction of compound 6 with the breast cancer protein (PDB ID: 1JNX) is shown in Figure 12. When the interaction of molecule **7** with the hCA II protein is examined in Figure 10, it is seen that the hydroxyl group in the molecule forms a hydrogen bond with the THR 200 protein. When the interaction of molecule **7** with the protein 1A52 in Figure 11 is examined, it is seen that molecule **7** creates hydrophobic interactions with the proteins around it. When the interaction of molecule **7** with the 1JNX protein is examined in Figure 12, it is seen that the carbonyl carbon in molecule **7** makes hydrogen bonds with the bound oxygen atom LYS 1702 and LEU 1701 proteins.

The high activity of these molecules does not mean that these molecules can be used as drugs. For this purpose, the movements of molecules in human metabolism are predicted by making ADME/T analysis of molecules, are given in Table 7. With these calculations, information about many properties of molecules is obtained. These calculated parameters are many chemical and biological properties of molecules. With this analysis, the movements of molecules in human metabolism are predicted. The entry of the molecule into human metabolism includes many processes including movements in metabolism and excretion from metabolism, these processes are called ADME/T, known as Absorption, Distribution, Metabolism, Excretion and Toxicity [21]. This analysis is divided into two. Firstly, the chemical properties of the molecules in the first part and the biological properties of the molecules in the second part are examined.

**Table 7:** ADME properties of molecules.

	**2**	**3**	**4**	**5**	**6**	**7**	**8**	**9**	**10**	**11**	**Referance range**
mol_MW	286	300	258	370	287	205	223	349	174	326	130–725
Dipole (D)	1.4	3.4	3.4	0.6	0.7	5.3	2.4	8.0	2.2	6.4	1.0–12.5
SASA	365	397	481	484	469	316	359	510	379	479	300–1000
FOSA	159	231	296	238	193	181	150	268	228	231	0–750
FISA	66	34	162	118	159	40	119	164	120	123	7–330
PISA	30	22	23	21	56	32	32	24	31	26	0–450
WPSA	109	111	0	106	61	63	58	54	0	100	0–175
Volume (A^3^)	589	653	828	834	776	511	573	909	612	822	500–2000
DonorHB	2	1	1	0	0	1	3	0	3	0	0–6
AccptHB	3.4	3.4	7.4	4	5	3.7	5.1	6	6.8	4	2.0–20.0
Glob (Sphere = 1)	0.9	0.9	0.9	0.9	0.9	1.0	0.9	0.9	0.9	0.9	0.75–0.95
QPpolrz (A^3^)	16.4	18.9	24.6	26.1	24.1	14.0	15.1	28.5	16.1	25.7	13.0–70.0
QPlogPC16	5.9	5.9	7.5	7.8	7.3	4.8	5.9	8.5	5.8	7.7	4.0–18.0
QPlogPoct	9.8	9.3	13.4	10.8	10.6	8.1	11.8	13.6	12.7	11.3	8.0–35.0
QPlogPw	7.1	5.5	10.0	5.4	6.8	5.9	10.4	7.5	11.9	5.4	4.0–45.0
QPlogPo/w	1.5	2.2	0.4	2.4	1.1	1.0	0.2	1.4	−0.3	2.3	−2.0–6.5
QPlogS	−2.0	−2.6	−1.9	−3.4	−2.4	−1.1	−1.4	−2.5	−1.1	−3.3	−6.5–0.5
CIQPlogS	−3.7	−4.0	−1.7	−5.2	−3.2	−1.9	−1.9	−3.8	−0.8	−4.2	−6.5–0.5
QPlogHERG	−2.8	−3.0	−3.5	−3.5	−3.9	−2.0	−2.8	−3.4	−3.0	−3.5	∗
QPPCaco (nm/sec)	2340	4669	285	748	307	4162	739	274	726	676	∗∗
QPlogBB	0.2	0.4	−1.1	−0.3	−0.8	0.4	−0.4	−0.9	−0.6	−0.4	−3.0–1.2
QPPMDCK (nm/sec)	4926	10000	127	1369	297	5088	741	243	350	1138	∗∗
QPlogKp	−2.4	−1.9	−4.0	−3.4	−4.1	−2.0	−3.3	−4.2	−3.2	−3.5	Kp in cm/h
IP (ev)	8.7	8.8	8.8	9.3	10.2	8.5	8.8	9.6	8.8	9.5	7.9–10.5
EA (eV)	0.5	0.7	0.2	1.0	1.0	0.7	0.1	0.9	0.0	0.8	−0.9–1.7
#metab	3	3	3	3	1	3	4	3	5	3	1–8
QPlogKhsa	−0.5	−0.3	−0.6	−0.1	−0.6	−0.6	−0.7	−0.5	−0.8	−0.2	−1.5–1.5
Human Oral Absorption	3	3	3	3	3	3	3	3	3	3	—
Percent Human Oral Abs.	96	100	73	92	78	100	79	79	76	91	∗∗∗
PSA	36	26	106	75	81	33	62	111	71	80	7–200
RuleOfFive	0	0	0	0	0	0	0	0	0	0	Maximum is 4
RuleOfThree	0	0	0	0	0	0	0	0	0	0	Maximum is 3
Jm	10.0	10.3	0.3	0.1	0.1	133.4	4.7	0.1	8.4	0.1	**—**

Abbreviations: #metab, number of likely metabolic reactions; accptHB, estimated number of hydrogen bonds that would be accepted by the solute from water molecules; CIQPlogS, conformation‐independent predicted aqueous solubility; dipole (D), dipol; donorHB, estimated number of hydrogen bonds that would be donated by the solute to water molecules; EA (eV), PM3 calculated electron affinity; FISA, hydrophilic component of the SASA; FOSA, hydrophobic component of the SASA; glob (Sphere =1) globularity descriptor; Human Oral Absorption, predicted qualitative human oral absorption; IP (ev), PM3 calculated ionization potential; Jm, predicted maximum transdermal transport rate; mol_MW, molecular weight; Percent Human Oral Absorption, predicted human oral absorption on 0% to 100% scale; PISA, *π* component of the SASA; PSA, Van der Waals surface area of polar nitrogen and oxygen atoms; QPlogBB, predicted brain/blood partition coefficient; QPlogHERG, predicted IC50 value for blockage of HERG K+ channels; QPlogKhsa, prediction of binding to human serum albumin; QPlogKp, predicted skin permeability; QPlogPC16, predicted hexadecane/gas partition coefficient; QPlogPo/w, predicted octanol/water partition coefficient; QPlogPoct, predicted octanol/gas partition coefficient; QPlogPw, predicted water/gas partition coefficient; QPlogS, predicted aqueous solubility; QPPCaco (nm/s), predicted apparent Caco‐2 cell permeability in nm/s; QPPMDCK (nm/s), predicted apparent MDCK cell permeability in nm/s; QPpolrz (A3), predicted polarizability; RuleOfFive, number of violations of Lipinski’s rule of five; RuleOfThree, number of violations of Jorgensen’s rule of three; SASA, total solvent accessible surface area (SASA); volume (A3), total solvent‐accessible volume; WPSA, weakly polar component of the SASA.

∗corcern below −5.

∗∗< 25 is poor and > 500 is great.

∗∗∗< 25% is poor and > 80% is high.

There are parameters that examine the chemical properties of molecules, which are many such as mol_MW (mole mass of molecules), dipole (dipole moment), SASA (solvent accessible surface area), volume (molecule volume), donorHB, and accptHB (number of hydrogen bonds that a molecule receives and gives off) parameter is calculated. On the other hand, there are many parameters that examine the biological properties of molecules, which are QPlogHERG (Predicted IC_50_ value for blockage of HERG K^+^ channels), QPPCaco and QPPMDCK (blood‐brain and blood‐bowel barriers), QPlogKp (Predicted skin permeability), QPlogKhsa (Prediction of binding to human serum albumin), and HumanOralAbsorption (Predicted qualitative human oral absorption). Apart from these, there are two parameters such as RuleOfFive [70, 71] and RuleOfThree [72] that examine the drug feasibility of molecules, which are known as RuleOfFive Number of violations of Lipinski’s rule of five and RuleOfThree is known as Number of violations of Jorgensen’s rule of three. The findings show that the numerical value of all these calculated parameters seems to have met all the requirements for human metabolism for studied molecule.

An examination of the data in the table reveals that the synthesized molecules largely meet the physicochemical and pharmacokinetic criteria for drug design. Their molecular weights range from 286 to 370 Da, consistent with Lipinski’s criteria. While 2, 5, and 6 have lower molecular weights, indicating more compact structures, 4, 8, and 9 have higher values, suggesting greater interaction potential at binding sites. The volume values are generally consistent with reference ranges, while the volumes of 7 and 9 were found to be relatively high.

Dipole moments range from 0.7 to 6.4 D. Molecules with lower dipole moments, such as 6 and 5, exhibit more hydrophobic properties, while 7 and 8, with their higher dipole moments, exhibit more polar behavior. Because most molecules have zero polar surface area, they have a high potential to cross biological membranes. The number of hydrogen bond donors ranges from 0 to 3, while the number of acceptors ranges from 3.4 to 7.4. 4, 9, and 10, in particular, have the potential to increase water solubility with their high acceptor counts.

When examining lipophilicity parameters, log*P* values are generally in the negative region, with hydrophilicity predominating. This is favorable for solubility, but excessively negative logP values can limit bioavailability. Solubility parameters, logS values, are around −2.6 to −3.4, indicating moderate solubility. LogBB values range from 0.2 to 0.4, suggesting a moderate likelihood of these molecules crossing the blood‐brain barrier.

When evaluating ADME parameters, oral absorption rates range from 73% to 100%. 2, 3, and 4 are the most advantageous molecules with 100% absorption rates. Values above 80% are generally quite suitable for drug candidacy. QPlogHERG values, which indicate cardiotoxicity, range from −2.8 to −3.6, indicating a low risk.

In terms of electronic properties, ionization potentials ranged from 8.3 to 9.6 eV, while electron affinity values remained mostly between 0 and 0.7 eV. This indicates that the compounds are chemically stable and their reactivity is not excessively high. In terms of their HOMO‐LUMO energy ranges, molecules 7, 8, and 9, with their high dipole moments, were found to be more noteworthy.

Generally speaking, molecules **3, 4**, and **8** stand out with their high absorption, favorable dipole moments, and strong ADME profiles. Molecules **2** and **6** are relatively disadvantaged due to their low dipole moments and limited hydrogen bonding capacity. Molecules **7** and **9**, on the other hand, offer stronger binding potential on protein surfaces due to their high dipole moments, sufficient absorption, and large volumes.

## 4. Conclusions

The hCA reaction contributes to many different pathological and physiological processes such as respiration, transport of CO_2_ and bicarbonate between metabolizing tissues and the lungs; pH and CO_2_ balancing; electrolyte secretion in different organs and tissues; biosynthetic reactions (such as gluconeogenesis, lipogenesis, and urea formation); osteoporosis; calcification and tumor formation [28, 35]. Many hCA inhibitors that participate in such activities have the potential to be used in the treatment of diseases such as glaucoma, obesity, edema, cancer, osteoporosis, and epilepsy by showing an inhibitory effect [28, 35]. In addition, the potential use of hCA inhibitors to combat infections caused by fungi, protozoa and bacteria has recently been the subject of new research [28, 35]. However, many systemic or ocular side effects can be observed with all CAIs. Therefore, the discovery of new CAs is very important.

Although most synthesized conduritols lacked anticancer activity in the tested models, compound 6 emerged as a promising candidate due to its selective cytotoxicity toward MCF‐7 breast cancer cells and induction of apoptosis. Together with its strong CA inhibition, this compound warrants further preclinical evaluation, while the broader scaffold may serve as a basis for future optimization. Flow cytometry studies also showed that when cells were applied at effective IC_50_ doses, breast cancer progressed to apoptosis within two processes. Our methyl‐substituted (halo/methoxy) conduritols behave as nonclassical CAIs, consistent with prior work showing that phenolic and coumarin scaffolds can inhibit CA without classical sulfonamide zinc binding and can be tuned for isoform selectivity [73]. In the anticancer context, broad reviews link CA inhibition to disruption of tumor metabolism and pH regulation, supporting our observation that CA blockade can lead to antiproliferative and pro‐apoptotic effects in breast cancer cells [54].

At the cytosolic isoform level (hCA I/II), triterpene–acetazolamide conjugates demonstrated potent hCA II inhibition together with cytotoxicity in human cancer cells, in line with our findings that strong I/II inhibition coincides with growth suppression [55]. Similarly, benzenesulfonamide–aroylhydrazone conjugates inhibited hCA I/II/IX/XII and induced apoptosis in MCF‐7 cells, paralleling our Annexin V apoptosis results [56]. Finally, sulfonamide s‐triazine hybrids screened across hCA I/II/IX/XII also showed activity against breast cancer models, reinforcing that mixed cytosolic/tumor‐associated CA inhibition can contribute to anticancer efficacy [74].

Overall, these parallels indicate that our conduritols belong to the emerging class of non‐sulfonamide CAIs with dual enzyme inhibition and anticancer potential. Extending future work to tumor‐associated isoforms (CA IX/XII) and combination studies in hypoxia models would strengthen their therapeutic relevance.

In antioxidant studies showed that the efficacy of syntheses **2, 6** and **10** as a result of DPPH and TAS studies. As a result of the FRAP study, it was determined that syntheses **2** and **10** showed high antioxidant power. In particular, it was determined that synthesis number **10** showed higher potency than Vitamin E and Vitamin C in all antioxidant studies. When compared to conventional chemicals, AZA, the methyl substituted mono‐ and dimethoxy conduritols were found to be significantly more effective in inhibiting some metabolic enzymes, hCA I and hCA II. The findings suggest that methyl substituted mono‐ and dimethoxy conduritols and derivatives (**1–10**) could serve as alternative candidates for the treatment of a number of diseases, including glaucoma, epilepsy, osteoporosis, gastric and duodenal ulcers, neurological disorders, and Alzheimer’s disease.

Experimental results were supported by docking tests, which demonstrated that there is a direct connection between the theoretical analysis and the experimental measurements. In accordance with its superior experimental inhibition constant, compound 7 demonstrated the lowest docking score for hCA II (–6.42 kcal/mol). Compound 6 also exhibited strong binding interactions with hCA I (–5.95 kcal/mol), which is also consistent with its superior experimental inhibition constant. The hypothesized inhibitory mechanism was further supported by the hydrogen bonding, *π*–*π* stacking, and halogen interactions that were predicted to occur at the catalytic site within the system. Molecular docking was found to be consistent with the experimental inhibition data, which meant that a structure–activity link was established across all of the series. These results verified that this was the case.

With a predicted human oral absorption above 80 percent, an acceptable log P (–2 to 2.5), and a low cardiotoxicity risk (QPlogHERG = –3), the ADME/T study revealed that all compounds matched the requirements established by Lipinski and Jorgensen. There were no violations in either Rule of Five or Rule of Three.

When taken as a whole, the results of both experiments and theoretical studies convincingly reveal that methyl‐substituted halogenated and methoxy conduritols constitute a unique class of non‐sulfonamide CAIs that exhibit dual antioxidant and anticancer activity [75–78]. Compounds 6, 7, and 10 in particular stand out as multifunctional leaders because they display highly effective enzyme inhibition, acceptable ADME/T profiles, and selective cytotoxicity.

In summary, this study makes a unique contribution by demonstrating that non‐sulfonamide, hydroxyl‐rich conduritol derivatives can simultaneously act as potent CA I/II inhibitors and exhibit anticancer activity in MCF‐7 breast cancer cells. Unlike classical sulfonamide CAIs, these scaffolds provide a distinct structural framework with the potential to reduce off‐target effects while maintaining high enzyme affinity. Among the synthesized compounds, compound 6 emerged as the strongest candidate, displaying selective cytotoxicity toward cancer cells, induction of apoptosis, and G0/G1 phase arrest. In addition, compound 10 showed superior antioxidant activity, and compound 8 achieved strong CA inhibition, further highlighting their multifunctional therapeutic potential. Collectively, these findings position methyl‐substituted conduritols as promising lead molecules for the development of next‐generation anticancer and enzyme‐targeting agents.

## Disclosure

All authors read and approved the final manuscript.

## Conflicts of Interest

The authors declare no conflicts of interest.

## Author Contributions

All authors contributed to the study conception and design. Material preparation, data collection and analysis were performed by Hayrani Eren Bostancı, Ebrar Büşra Yıldırım, Feyza Nur Çetin, Dilek Kaplan, Ümit Muhammed Koçyiğit, Alireza Poustforoosh, Burak Tüzün, and Latif Kelebekli. The first draft of the manuscript was written by Hayrani Eren Bostancı and all authors commented on previous versions of the manuscript.

## Funding

This study was supported by Scientific Research Project Fund of Sivas Cumhuriyet University (CUBAP), RGD‐020 ECZ079.

## Supporting information


**Supporting Information** Additional supporting information can be found online in the Supporting Information section.. The supplementary data include detailed synthetic procedures, additional spectroscopic characterization, and the NMR spectra of the compounds 2, 3, 4, 5, 6, 7, 8, 9, 10, and 11. These data provide further validation of the experimental and findings discussed in the main text.

## Data Availability

The datasets generated during and/or analyzed during the current study are available from the corresponding author on reasonable request.
