# Supplementary material for: Investigation of Carbonic Anhydrase Inhibition, Antioxidant Properties, and Selective Anticancer Activity of Methyl‐Substituted Halogenated and Methoxy Conduritols
Source: Biomed Res Int. 2026 Feb 12;2026:5819417. doi: 10.1155/bmri/5819417 (PMC12900579; doi:10.1155/bmri/5819417)
Supplement: Supplementary file 1 — Supporting Information Additional supporting information can be found online in the Supporting Information section.. The supplementary data include detailed synthetic procedures, additional spectroscopic characterization, and the NMR spectra of the compounds 2, 3, 4, 5, 6, 7, 8, 9, 10, and 11. These data provide further validation of the experimental and findings discussed in the main text. [file BMRI-2026-5819417-s001.docx]

**Supplementary information**

**EXPERIMENTAL SECTION**

**General Information**

A capillary melting apparatus (electrothermal) was used for determination of melting points and are the results are presented without correction. IR spectra were taken with a Shimadzu Spectrophotometer device. The ^1^H NMR, ^13^C NMR spectra were recorded on 400 (100) MHz Bruker spectrometer (Avance III) and are reported in *δ* units with TMS as internal standard. All compounds were characterized with ^1^H and ^13^C NMR spectra. TLC was performed on E. Merck Silica Gel 60 F_254_ plate (0.2 mm). Flash-column chromatography was performed on Merck silica gel (60 mesh). Organic solvents were purified by distillation. All organic extracts were dried with MgSO4, filtered, and concentrated on a rotary evaporator. HRMS were recorded by LC-MS TOF electrospray ionization technique (6230, Agilent).

**(±)-5,6-Dibromo-2-methylcyclohex-2-ene-1,4-diol (2):**

Prepared according to the procedure described in the literature.^1^ (19 g, 85%) (recrystallized from AcOEt/hexane), mp 111-112 ^o^C; ^1^H-NMR (400 MHz, CD_3_OD) δ 5.48 (dd, 1H, *J* = 1.7, 3.3 Hz, -CH=C), 4.20-4.39 (m, 2H, -OH), 4.06-4.18 (m, 2H, -CHBr), 1.79-1.81 (m, 3H, -CH_3_); ^13^C-NMR (100 MHz, CD_3_OD) δ 137.4 (C=C), 125.9 (C=C), 75.4 (C-O), 72.7 (C-O), 60.3 (C-Br), 60.2 (C-Br), 17.9 (-CH_3_); IR (KBr, cm^-1^): 3390, 3352, 2974, 2908, 1678, 1400, 1284, 1161, 1060, 1041, 991, 956, 840, 694, 547.

**(±)-3,6-Dimethoxy-4-methylcyclohex-4-ene-1,2-diol (3):**

Prepared according to the procedure described in the literature.^2^ (0.96 g, 76%); mp 159-161 ^o^C (recrystallized from CH_2_Cl_2_/Et_2_O); ^1^H-NMR (400 MHz, CDCl_3_) δ 5.37 (d, 1H, *J* = 1.3 Hz, -CH=C), 5.01 (d, 1H, *J* = 4.8 Hz, -COH), 4.96 (d, 1H, *J* = 4.8 Hz, -COH), 3.58-3.56 (m, 1H, -CHOC), 3.26-3.20 (m, 1H, CHOC), 3.37 (s, 3H, -OCH_3_), 3.32 (s, 1H, -OCH_3_), 3.36 (s, 1H, -OH), 2.51-2.50 (m, 3H, -OH), 1.65 (s, 3H, -CH_3_); ^13^C-NMR (100 MHz, CD_3_OD) δ 135.8 (C=C), 124.1 (C=C), 83.8 (C-OCH_3_), 81.2 (C-OCH_3_), 74.8 (C-O), 74.5 (C-O), 58.3 (-OCH_3_), 57.1 (-OCH_3_), 19.1 (CH_3_); IR (KBr, cm^-1^): 3390, 3352, 2974, 2908, 1678, 1400, 1284, 1161, 1060, 1041, 991, 956, 840, 694, 547; HRMS: m/z calculated for C_9_H_16_NaO_4_ [M+Na]^+^, 211.0941; found: 211.0970.

**(±)-3,6-Dimethoxy-4-methylcyclohex-4-ene-1,2-diyl diacetate (4):**

Prepared according to the procedure described in the literature.^2^ (7.2 g, 89%); Compound **4** was recrystallized from CH_2_Cl_2_/hexane to give colorless crystals, mp 66-68 ^o^C; ^1^H-NMR (400 MHz, CDCl_3_): δ 5.50-5.46 (m, 1H, -CH=C), 5.22 (dd, 1H, A part of AB system, *J* = 7.8, 11.0 Hz, -CHOAc), 5.11 (dd, 1H, B part of AB system, *J* = 7.8, 11.0 Hz, -CHOAc), 4.02-3.92 (m, 2H, -CH-O), 3.31 (s, 3H, -OCH_3_), 3.30 (s, 3H, -OCH_3_), 2.03 (s, 3H, -OAc), 2.02 (s, 3H, -OAc), 1.74 (s, 3H, -CH_3_); ^13^C-NMR (100 MHz, CDCl_3_): δ 170.2 (C=O), 170.1 (C=O), 136.2 (C=C), 123.8 (C=C), 80.9 (C-O), 78.2 (C-O), 72.3 (C-O), 71.9 (C-O), 56.9 (-OCH_3_), 56.4 (-OCH_3_), 29.7 (-CH_3_), 20.9 (-CH_3_), 18.6 (-CH_3_); IR (KBr, cm^-1^): 2951, 1748, 1435, 1366, 1238, 1215, 1096, 1033, 957, 880, 736, 698, 651, 601, 571; HRMS: m/z calculated for C_13_H_20_NaO_6_ [M+Na]^+^, 295.1152; found: 295.1211.

**(±)-5,6-Dibromo-2-methylcyclohex-2-ene-1,4-diyl diacetate (5):**

Prepared according to the procedure described in the literature.^2^ (11.15 g, 86%); Compound **5** was recrystallized from EtOH as colorless crystals, mp 94-96 ^o^C; ^1^H-NMR (400 MHz, CDCl_3_): δ 5.84-5.79 (m, 1H, H_4_), 5.70-5.64 (m, 1H, H_1_), 5.53-5.49 (m, 1H, H_3_), 4.32-4.24 (m, 2H, -CHBr), 2.19 (s, 3H, -COCH_3_), 2.14 (s, 3H, -COCH_3_), 1.67 (s, 3H, -CH_3_); ^13^C-NMR (100 MHz, CDCl_3_): δ 170.0 (C=O), 169.9 (C=O), 136.2 (C=C), 123.8 (C=C), 74.8 (C-O), 73.6 (C-O), 53.4 (C-Br), 52.6 (C-Br), 20.9 (-CH_3_), 20.7 (-CH_3_), 18.8 (-CH_3_); IR (KBr, cm^-1^): 2978, 1724, 1435, 1369, 1228, 1169, 1123, 1088, 1053, 1022, 995, 910, 883, 772, 702;

**2-Bromo-5-methyl-1,4-phenylene diacetate (6):**

Prepared according to the procedure described in the literature.^2^ (0.62 g, 80%) as colorless crystals, mp 123-125 ^o^C; ^1^H-NMR (400 MHz, CDCl_3_): δ 7.31 (br s, 1H, H_3_ or H_6_), 7.03 (br s, 1H, H_3_ or H_6_), 2.36 (s, 3H, -OAc), 2.33 (s, 3H, -OAc), 2.16 (s, 3H, -CH_3_); ^13^C-NMR (100 MHz, CDCl_3_): δ 168.7 (C=O), 168.6 (C=O), 147.2 (arom-quaternary), 145.8 (arom-quaternary), 131.1 (arom-CH_3_), 126.5 (arom.), 125.4 (arom.), 112.9 (arom-Br), 20.8 (-CH_3_), 20.7 (-CH_3_), 16.1 (-CH_3_); IR (KBr, cm^-1^): 2939, 1744, 1485, 2369, 1204, 1142, 1007, 918, 867, 810, 652, 586; HRMS: m/z calculated for C_13_H_20_O_6_ [M+H]^+^, 286.9914; found: 286.9882.

**(±)-2-Bromo-5-methyl-7-oxabicyclo[4.1.0]hept-4-en-3-ol (7):**

Prepared according to the procedure described in the literature.^2,3^ (4.30 g, 70%). The product **7** was recrystallized from AcOEt/hexane as colorless crystals, mp 128-130 ^o^C; ^1^H-NMR (400 MHz, CDCl_3_): δ 5.62 (dd, 1H, *J* = 1.8, 3.8 Hz, CH=C), 4.49-4.43 (m, 1H, -CHO-), 4.02 (dd, 1H, *J* = 1.2, 8.4 Hz, -CHBr), 3.74 (dd, 1H, *J* = 0.8, 4.0 Hz, -CHO-), 3.36 (dd, 1H, *J* = 2.4, 4.0 Hz, -CHO-), 2.47 (d, 1H, *J* = 4.3 Hz, -OH), 1.96 (dd, 1H, *J* = 1.7, 2.6 Hz, -CH_3_); ^13^C-NMR (100 MHz, CDCl_3_): δ 132.2 (C=C), 127.5 (C=C), 70.9 (C-O), 56.0 (C-Br), 55.6 (C-O), 55.5 (C-O), 21.1 (-CH_3_); IR (KBr, cm^-1^): 3318, 3252, 3024, 2978, 2916, 1663, 1450, 1381, 1339, 1273, 1234, 1180, 1185, 1034, 1011, 944, 899, 800, 822, 756, 656, 611; HRMS: m/z calculated for C_7_H_9_KO_2_ [M+K]^+^, 242.9418; found: 243.0123.

**(±)-3-Bromo-6-methylcyclohex-5-ene-1,2,4-triol (8):**

Prepared according to the procedure described in the literature.^2^ (2.3 g, 51%). The product **8** was recrystallized from EtOH as colorless crystals, mp 132 ^o^C; ^1^H-NMR (400 MHz, CD_3_OD): δ 5.55 (dd, 1H, *J* = 1.6, 4.0 Hz, CH=C), 4.26 (dt, 1H, *J* = 2.2, 8.4 Hz, H_4_), 4.01 (dd, 1H, *J* = 1.2, 8.4 Hz, H_1_), 3.72 (dd, 1H, *J* = 0.8, 4.0 Hz, H_2_), 3.39 (dd, 1H, *J* = 2.4, 4.0 Hz, H_3_), 1.96 (dd, 3H, *J* = 1.6, 2.6 Hz, -CH_3_); ^13^C-NMR (100 MHz, CD_3_OD): δ 132.1 (C=C), 128.6 (C=C), 70.3 (C-O), 55.7 (C-O), 55.2 (C-O), 54.6 (C-Br), 19.6 (-CH_3_); IR (KBr, cm^-1^): 3317, 3240, 1450, 1338, 1273, 1234, 1180, 1010, 948, 898, 860, 821, 756, 655, 601, 547; HRMS: m/z calculated for C_13_H_20_NaO_6_ [M+Na]^+^, 244.9784; found: 244.9766.

**(±)-3-Bromo-6-methylcyclohex-5-ene-1,2,4-triyl triacetate (9):**

Prepared according to the procedure described in the literature.^2^ (1.74 g, 77%). Compound **9** was recrystallized from AcOEt/hexane as colorless crystals, mp 86-88 ^o^C; ^1^H-NMR (400 MHz, CDCl_3_): δ 5.65-5.62 (m, 1H, CH=C), 5.55 (dd, 2H, *J* = 2.8, 6.0 Hz, -CHOAc), 5.30 (dd, 1H, *J* = 2.8, 6.0 Hz, -CHOAc), 4.36 (dd, 1H, *J* = 2.8, 5.6 Hz, -CHBr-), 2.14 (s, 3H, -COCH_3_), 2.13 (s, 6H, -COCH_3_), 1.74 (s, 3H, -CH_3_); ^13^C-NMR (100 MHz, CDCl_3_): δ 170 (C=O), 169.9 (C=O),169.8 (C=O), 136.3 (C=C), 122,6 (C=C), 71.4 (C-O), 71.1(C-O), 70.8 (C-O), 47.7 (C-Br), 20.9 (-CH_3_), 20.8 (-CH_3_), 20.7 (-CH_3_), 19.4 (-CH_3_); IR (KBr, cm^-1^): 2322, 1736, 1520, 1435, 1369, 1219, 1157, 1096, 1022, 976, 910, 860, 775, 663, 509, 451;

**(±)-6-Methoxy-4-methylcyclohex-4-ene-1,2,3-triol (10):**

Prepared according to the procedure described in the literature.^2^ (1.16 g, 93%); mp 63-65 ^o^C (recrystallized from EtOH); ^1^H-NMR (400 MHz, CDCl_3_) δ 5.4-5.42 (m, 1H, CH=C), 5.15-5.09 (m, 1H, -CHO-), 4.66-5.61 (m, 1H, -CHO-), 4.45-4.44 (m, 1H, -CHO-), 4.05 (s, 1H, -OH), 3.82 (s, 1H, -OH), 3.65-3.56 (m, 1H, -CHOMe), 3.46 (s, 3H, -OCH_3_), 2.71 (s, 1H, -OH), 1.79 (s, 3H, -CH_3_); ^13^C-NMR (100 MHz, CDCl_3_) δ 137.2 (C=C), 121.2 (C=C), 81.2 (C-O), 75.9 (C-O), 74.2 (C-O), 73.9 (C-O), 56.8 (-OCH_3_), 18.7 (-CH_3_); IR (KBr, cm^-1^): 3316, 2974, 2903, 2877, 1454, 1371, 1321, 1283, 1194, 1088, 1051, 995, 966, 891, 825, 611, 521; HRMS: m/z calculated for C_8_H_14_NaO_4_ [M+Na]^+^, 197.0785; found: 197.0874.

**(±)-2-bromo-6-chloro-5-methylcyclohex-4-ene-1,3-diyl diacetate (11)**

Prepared according to the procedure described in the literature.^3^ as colorless oil (0.66 g, 56 %); ^1^H-NMR (400 MHz CDCl_3_ ppm): δ 5.58 (br d, 1H, *J* = 7.6 Hz, H_3_), 5.49-5.52 (m, 1H, H_4_), 5.39 (dd,1H, *J* = 3.6, 2.4 Hz, H_1_), 4.49 (dd, 1H, *J* = 7.6, 2.4 Hz, H_2_), 4.34 (d,1H, *J* = 3.6 Hz, H_6_), 2.11 (s, 3H, -OAc), 2.09 (s, 3H, -OAc), 1.82 (s, 3H, -CH_3_); ^13^C-NMR (100 MHz CDCl_3_ ppm): δ 170.1 (C=O), 169.7 (C=O), 135.3 (C quaternary), 123.6 (C=C), 74.8 (-O-C), 71.7 (-O-C), 57.3 (-C-Br), 46.2 (-C-Cl), 20.9 (-CH_3_), 20.7 (-CH_3_), 20.6 (-CH_3_); IR (KBr, cm^-1^): 2359, 2324, 1746, 1373, 1223, 1085, 1035, 862, 788; HRMS: m/z calculated for C_11_H_14_BrClNaO_4_ [M+Na]^+^ (Br^79^, Cl^35^), 346.9656; found: 346.9692 and C_11_H_14_BrClNaO_4_ [M+Na]^+^ (Br^81^, Cl^35^ or Br^79^, Cl^37^), 348.9636; found: 348.9670.

**References:**

1. Leermann, T., Block, O., Podeschwa, M. A. L., Pfüller, U., & Altenbach, H. (2010). *De novo* synthesis and lectin binding studies of unsaturated carba-pyranoses. Organic & Biomolecular Chemistry, 8, 3965-3974.
2. Kelebekli, L., & Kaplan, D. (2017). Stereospecific synthesis of novel methyl-substituted mono-and di-methoxy conduritols. Tetrahedron, 73(1), 8-13.
3. Kelebekli, L., & Kaplan, D. (2020). A novel and stereoselective synthesis of 2-bromo-6-chloro-5-methylcyclohex-4-ene-1, 3-diyl diacetate: conduritol-A derivative. Journal of the Iranian Chemical Society, 17, 803-808.

**NMR spectra of the compounds 2,3, 4, 5, 6, 7, 8, 9, 10 and 11**

**Figure S1**. 400 MHz ^1^H-NMR spectra of compound **2** (CD_3_OD)

**Figure S2**.100 MHz ^13^C-NMR spectra of compound **2** (CD_3_OD)

**Figure S3**. 400 MHz ^1^H-NMR spectra of compound **3** (CDCl_3_)

**Figure S4**. 100 MHz ^13^C-NMR spectra of compound **3** (CD_3_OD)

**Figure S5**. 400 MHz ^1^H-NMR spectra of compound **4** (CDCl_3_)

**Figure S6**. 100 MHz ^13^C-NMR spectra of compound **4** (CDCl_3_)

**Figure S7**. 400 MHz ^1^H-NMR spectra of compound **5** (CDCl_3_)

**Figure S8**. 100 MHz ^13^C-NMR spectra of compound **5** (CDCl_3_)

**FigureS9**. 400 MHz ^1^H-NMR spectra of compound **6** (CDCl_3_)

**Figure S10**. 100 MHz ^13^C-NMR spectra of compound **6** (CDCl_3_)

**Figure S11**. 400 MHz ^1^H-NMR spectra of compound **7** (CDCl_3_)

Figure S12. 100 MHz ^13^C-NMR spectra of compound **7** (CDCl_3_)

**Figure S13**. 400 MHz ^1^H-NMR spectra of compound **8** (CD_3_OD)

**Figure S14**. 100 MHz ^13^C-NMR spectra of compound **8** (CD_3_OD)

**Figure S15**. 400 MHz ^1^H-NMR spectra of compound **9** (CDCl_3_)

**Figure S16**. 100 MHz ^13^C-NMR spectra of compound **9** (CDCl_3_)

**Figure S17**. 400 MHz ^1^H-NMR spectra of compound **10** (CDCl_3_)

**Figure S18**. 100 MHz ^13^C-NMR spectra of compound **10** (CDCl_3_)

**Figure S20**. 400 MHz ^1^H-NMR spectra of compound **11** (CDCl_3_)

**Figure S21**. 100 MHz ^13^C-NMR spectra of compound **11** (CDCl_3_)
